# Improved Cassava Varieties and Smallholder Farmers' Productivity and Welfare in Ghana: Insights From a Robust Impact Estimator Selection Approach

**DOI:** 10.1002/pei3.70186

**Published:** 2026-07-17

**Authors:** Kodjo Kondo, Bright Owusu Asante, Omphile Temoso, Stephen Prah

**Affiliations:** ^1^ AGRA, CSIR Office Complex Accra Ghana; ^2^ Department of Agricultural Economics, Agribusiness and Extension Kwame Nkrumah University of Science and Technology Kumasi Ghana; ^3^ UNE Business School University of New England Armidale New South Wales Australia; ^4^ Department of Agricultural and Resource Economics North Carolina State University Raleigh North Carolina USA

**Keywords:** adoption, cassava improved varieties, endogenous treatment effect, Ghana, propensity score matching

## Abstract

The development and dissemination of improved agricultural technologies are crucial for enhancing productivity and the welfare of smallholder farmers. Although there is growing empirical evidence on the impact of improved agricultural technologies on various economic and social outcomes, some studies often overlook potential biases. Stemming from both observable and unobservable characteristics, these biases can result in misleading conclusions. To fill the gap, this study examined the impact of the adoption of improved cassava varieties (ICV) on farm performance and household welfare using a sample of 608 households from 14 rural communities in Ghana. To account for potential biases, the study used appropriate matching algorithm, local average treatment effects and endogenous treatment effects regression. The findings show that ICV adoption has a positive impact on cassava yields, whole‐farm land productivity, and household crop incomes. We also show that adopters have significantly lower total annual per capita and food expenditures than non‐adopters. Furthermore, adopters were found to spend more on children's education. Thus, policies and strategies aimed at increasing ICV adoption rates could lead to increased land productivity and crop incomes, as well as enhanced food security and higher investment in children's education, particularly for female‐headed farm households.

## 1 Introduction

Global food insecurity remains a pressing development challenge. Recent United Nations data (UN 2023) reveal that 2.4 billion people experience moderate food insecurity and an additional 900 million suffer severe food insecurity, casting doubt on progress toward Sustainable Development Goals 1 and 2. The situation is further compounded by the COVID‐19 pandemic, the Russia‐Ukraine conflict, Iran‐Israel conflicts, and climate change, all of which intensify food insecurity, particularly in sub‐Saharan Africa (SSA) (UN 2023; FAO 2024). The Food and Agriculture Organization (FAO 2024) highlights that 45 countries currently require urgent external food aid, 33 of which are in Africa.

Addressing food insecurity in SSA requires a sustained focus on agricultural productivity, where the region consistently records the lowest performance globally (Gatti et al. 2023; Ongoma et al. 2025; Bentze and Wollburg 2025; Ritchie 2022). Cassava is emblematic of this challenge. As a staple food and major income source for millions of smallholder households, cassava holds significant potential for food security and poverty reduction. However, productivity remains far below potential. For instance, in Ghana, cassava yields an average of 21 metric tonne per hectare against a potential of 49 tonne per hectare (Ministry of Food and Agriculture 2024; Acheampong et al. 2022). This gap reflects broader constraints including low adoption of improved agricultural technologies, limited access to input and output markets, credit, and extension services (Acheampong et al. 2022).

To close this productivity gap, governments and development organizations have promoted improved cassava varieties (ICVs) across SSA. In Ghana, programs such as the World Bank funded West Africa Agricultural Productivity Programme (WAAPP), the International Fund for Agricultural Development (IFAD) funded Root and Tuber Improvement Project (RTIMP), and the Government of Ghana flagship programme on Planting for Food and Jobs (PFJ) have supported the dissemination of improved cassava varieties to smallholder farmers. Despite this investment, rigorous evidence on whether ICV adoption translates into measurable welfare improvements among Ghanaian smallholders remains scarce.

The existing literature (Acheampong et al. 2022; Mdoda et al. 2025; Kamara et al. 2025; Ahimbisibwe et al. 2023; Muhaimin et al. 2020; Bosompem et al. 2024; Ossai et al. 2025; Yi et al. 2026) documents positive impacts of ICV adoption across several African contexts. For instance, Ahimbisibwe et al. (2023) observed an increase in productivity, income, and consumption expenditure in Uganda due to ICVs. Similarly, Ayanwale et al. (2026) reported an increase in cassava yield per hectare in Nigeria as a result of ICV adoption. Muhaimin et al. (2020) found that ICVs positively impacted agricultural income and food security. In Tanzania, Kinuthia and Mabaya (2017) found that ICVs led to higher yields. Mwebaze et al. (2024) examined cassava adoption among 1200 smallholder farmers in Malawi, Tanzania, and Uganda, and found that varietal characteristics such as yield and early maturity were critical drivers of adoption, and that access to extension and credit increased the attractiveness of yield‐improving traits. Similarly, using an endogenous switching regression approach, Kamara et al. (2025) found that adoption of improved crop varieties significantly increased productivity and net income among smallholder farmers in Nigeria, while Mdoda et al. (2025) reported comparable productivity and efficiency gains from improved crop variety adoption among smallholder farmers in South Africa.

However, these findings are context‐specific and may not generalize to Ghana due to differences in institutional environments, market access, extension systems, and farmer characteristics. Ghana's cassava production remains relatively underrepresented in the rigorous impact evaluation literature, particularly regarding the welfare effects of ICV adoption. This gap is important because Ghana provides a distinct institutional and production context, where cassava technology dissemination has been strongly influenced by public agricultural interventions such as the WAAPP and other root and tuber development initiatives. Differences in extension systems, input markets, and institutional arrangements imply that evidence from other African countries may not fully capture the impacts of ICV adoption in Ghana. Furthermore, despite cassava's importance as a staple food, income source, and climate‐resilient crop for smallholder farmers, limited evidence exists on whether improved cassava varieties generate broader welfare improvements, including farm income, household expenditure, and gender‐specific economic outcomes. Therefore, this study evaluates the impact of ICV adoption on the productivity and welfare of smallholder farmers in Ghana using data collected from the WAAPP programme and provides context‐specific evidence on the effectiveness of agricultural technology interventions.

Despite the growing body of empirical evidence on the impact of ICVs adoption on smallholder farmers' economic and social outcomes, there are still some limitations and unanswered questions in the literature. A key limitation of the current research is the absence of a suitable counterfactual and the issue of selection bias and unobserved heterogeneity. Most studies fail to adequately address selection bias and unobserved heterogeneity; thus, they are fundamental threats to credible causal inference in non‐experimental settings (Morgan and Winship 2014; Mendola 2007). While Awotide et al. (2015) applied both Propensity Score Matching (PSM) and instrumental variables (IV) to address observable and unobservable confounding, PSM estimators can lose robustness when the Conditional Independence Assumption is violated (Rosenbaum 2002).

The contributions of the study are as follows. First, we examine the impacts of ICVs on productivity, household income and expenditure in Ghana. This provides an understanding of ICVs adoption in enhancing agricultural productivity, empowering smallholder farmers, and improving living standards. This research not only highlights the benefits for ICVs to alleviate poverty and increase food security but also provides valuable insights for policymakers to develop targeted interventions that promote sustainable agricultural practices and resilience in the face of economic challenges.

Second, we utilize the average treatment effect on the treated (ATT) and the local average treatment effect (LATE) to estimate the impacts of ICVs adoption on productivity, household income, and expenditures. Unlike Awotide et al. (2015), we applied well‐balanced PSM estimators and tested them against two specifications of endogenous treatment‐effects regressions. By considering productivity, farm income, and expenditure levels as indicators, we provide a more comprehensive assessment of household welfare. This is due to the positive correlation between increase in productivity, incomes, and expenditure levels, and the incidence of poverty and food insecurity (Feleke et al. 2016; Wossen et al. 2019).

Third, we disaggregated the impacts of ICVs adoption on female household income in Ghana. This offers a holistic understanding of the role of women in agricultural productivity and economic development, providing insights into how ICV adoption can reduce gender disparities. Also, the findings will inform targeted policies and programs that support women in agriculture, promoting equitable growth and fostering sustainable livelihoods within communities.

Fourth, the study adds to burgeoning adoption studies in Africa by providing nuanced insights into IVCs adoption and subsequent impacts on productivity and household welfare. By focusing on specific contexts, such as Ghana, we enrich the understanding of local agricultural dynamics and offer empirical evidence that highlights both the challenges and successes of ICV adoption. Lastly, the findings will offer insights for policymakers to support continued investment in the dissemination and promotion of ICVs for smallholder farmers. This not only lays a solid foundation for further investigation into their impact but also paves the way for their expansion. Such initiatives are critical to improving food security and reducing poverty in developing countries.

The remainder of the paper is organized into five sections. Section 2 reviews the theoretical framework of quasi‐experimental impact evaluation methods and empirical models. Section 3 provides a description of the data collection process and the estimation variables. Section 4 shows the empirical results and discussion, while Section 5 provides the conclusion and recommendations.

## 2 Theoretical Framework

This section presents a review of PSM and IV methods, as well as their use in assessing the causal effect of ICVs adoption on land productivity, incomes, and expenditures.

### 2.1 Counterfactual and Impact Evaluation

To distinguish the effect of ICVs adoption on outcome indicators from other influencing factors, it is necessary to estimate the counterfactual. We accomplish this by employing the average treatment effect on the treated (ATT). ATT represents the difference between the mean outcome of adopters and the hypothetical mean outcome of the same adopters had they not received the treatment. This is specified as follows:

(1) $$\mathrm{ATT}=E\left(Y_{1i}|d_{i}=1\right)-E\left(Y_{1i}|d_{i}=0\right)$$

where *Y*_*i*_ is one of the outcome variables of interest for household *i*; *d*_*i*_ is the ICV adoption categorical variable; $E\left(Y_{1i}|d_{i}=1\right)$ denotes the average outcome of the adopters; and $E\left(Y_{1i}|d_{i}=0\right)$ is the unobserved counterfactual.

Valid impact evaluation requires a credible counterfactual that accounts for both observed and unobserved differences between adopters and non‐adopters. Failure to do so yields biased ATT estimates (Caliendo and Kopeinig 2008; Winters et al. 2010). Impact evaluation methods fall into two broad categories: experimental and quasi‐experimental (Shahidur et al. 2016). Randomized controlled trials (RCTs) are the gold standard, as random assignment produces statistically equivalent treatment and control groups (Baker 2000), but they are costly, ethically constrained, and difficult to implement in practice (Cavatassi et al. 2011). Quasi‐experimental methods including PSM, IV, difference‐in‐differences, and regression discontinuity are therefore commonly employed to construct valid counterfactuals (Shadish et al. 2002). Given the cross‐sectional nature of the data and ex‐post evaluation design, the study relied on PSM and IV.

### 2.2 Propensity Score Matching

PSM pioneered by Rosenbaum and Rubin (1983), is the most popular non‐experimental method used in impact studies of agricultural‐related interventions (e.g., Awotide et al. 2015; Ricome et al. 2024; Selorm et al. 2023; Wossen et al. 2019). PSM uses estimated probabilities of adoption, known as propensity scores, derived from a binary choice model (logit or probit). It is fitted using a carefully selected set of pre‐treatment and time‐invariant observed covariates. The goal is to match adopters with their most similar non‐adopters based on these propensity scores. Most studies [e.g., Awotide et al. 2015; Bravo‐Ureta et al. 2021; González‐Flores et al. 2014; Villano et al. 2015] use PSM as a first stage to address observed heterogeneity. First, PSM effectively reduces selection bias by balancing observable characteristics between treated and control groups, thereby minimizing confounding variables. This makes it a practical initial step in causal analysis. Second, the PSM is relatively straightforward to implement and understand, allowing researchers to match individuals based on observable traits, which aids in interpreting results. Furthermore, using PSM serves as a preliminary method that sets the stage for more complex analyses; once matched samples are created and hidden biases are controlled as outline by Rosenbaum and Rubin (1983), we utilize additional techniques, such as instrumental variables (IV) or endogenous treatment regression to further address unobserved heterogeneity. We used a probit model to estimate the propensity scores, expressed as:

(2) $$\left(d_{i}=1|X_{\mathrm{ji}}\right)=P\left(\alpha_{j}X_{\mathrm{ji}}+w_{i}>0\right)$$

where $\alpha_{j}$ is the coefficient associated with the *j*th covariate; *X*_*j*_ are pre‐treatment and time‐invariant covariates unaffected by adoption; and *w*_*i*_ denotes the random error assumed to be normally distributed [*w*_*i*_ ~ *N* (0, 1)].

PSM estimators include one‐to‐one nearest neighbor matching (NNM), k‐NNM, kernel‐based matching (KBM), radius matching (RM), and local linear regression matching (LLRM) (Becker and Ichino 2002; Leuven and Sianesi 2003), with covariate‐based matching (Abadie et al. 2004) used to check robustness. While all algorithms should yield equivalent results in principle, trade‐offs between bias and efficiency arise in practice (Caliendo and Kopeinig 2008). Match quality is assessed using the *t*‐test of mean differences (Rosenbaum and Rubin 1985), Rubin's *B* and *R* statistics where *B* < 25 and 0.5 < *R* < 2 indicate sufficient balance (Rubin 2001), and Sianesi's (2004) likelihood‐ratio test, which should yield a smaller pseudo‐*R* ^2^ and insignificant *p*‐values after matching. The PSM further rests on two key assumptions: the conditional independence assumption (CIA) and the common support assumption, the latter was enforced via 2% trimming (Smith and Todd 2005). To test the sensitivity of the estimated ATT to hidden bias, we instrument for ICV adoption using extension agents (EAs) and innovation platforms (IPs). The relevance condition is satisfied because EAs establish demonstration plots and distribute planting materials, while IPs coordinate dissemination networks, thus, both directly targeting adoption decisions (Di Falco et al. 2011; Adam and Abdulai 2023; Kubitza and Krishna 2020). The exclusion restriction requires that these instruments affect productivity and welfare only through ICV adoption, not independently. We argue this holds for two reasons. First, in the WAAPP‐Ghana context, EAs and IPs were deployed specifically as ICV dissemination mechanisms. Their primary mandate was technology promotion, not generalized agronomic training or input provision, and contact with farmers was structured around variety trials and planting material distribution, meaning any productivity effect operates through the adoption channel. Second, conditional on adoption status, a farmer's exposure to an EA or IP provides no additional agronomic advantage; thus, the knowledge and materials transferred are embodied in the improved variety itself. As a robustness check, we report overidentification tests and discriminate between the IV estimates and the PSM results based on the violation of the CIA or not (see Section 4.2).

### 2.3 Linear Regression With Endogenous Treatment Effects

The linear regression with endogenous treatment effects is a variant of the IV approach with a binary treatment variable. The model is also known as endogenous dummy variable models (Heckman 1978) or endogenous treatment‐regression models (StataCorp 2015a) and uses Ordinary Least Square (OLS) regression for the outcome variable and a probit model to predict the deviation from the CIA imposed in PSM. There are two main specifications of the endogenous treatment‐regression models according to the assumption regarding the error term in the outcome model. The model is regarded as constrained, restricted, or reduced when the error term in the outcome model is assumed to be identical for the adopters and non‐adopters, while the unconstrained specification distinguishes separate error terms for the potential‐outcome models for adopters and non‐adopters.

#### 2.3.1 The Restricted Model Specification

Following Wooldridge (2010), Greene (2012) and StataCorp (2015a), we expressed the reduced form of the endogenous treatment effect regression model as:

(3) $$Y_{i}=\beta_{j}X_{\mathrm{ji}}+\delta d_{i}+e_{i}$$

(4) $$d_{i}^{*}=\alpha_{k}Z_{k}+w_{i}$$

(5) $$d_{i}=\left\{\begin{array}{c} 1,\text{if}\;d_{i}^{*}>0\;\\ 0,\text{otherwise}\; \end{array}\right.$$

where *Y*_*i*_ denotes the outcome variables defined earlier; $X_{j}$ is a vector of covariates that are also used in the PSM estimators; $d_{i}^{*}$ denotes the unobserved latent variable for the adoption decision, which really occurs under conditions in Equation (5), and the adoption indicator variable is assumed to be endogenous (i.e., correlated with the error term ei); *δ* in Equation (3) is the biased impact of ICVs adoption on the outcome variable under violation of the CIA; and *Z*_*k*_ is a vector of instruments.

We assume that

(6) $$\mathrm{cov}\;\left(x_{i},e_{i}\right)=\mathrm{cov}\left(z_{i},e_{i}\right)=0$$

(7) $$\mathrm{cov}\;\left(w_{i},e_{i}\right)=\rho$$

where ρ(rho) is the correlation coefficient between the two error terms ei and wi that are assumed to follow a bivariate normal distribution with mean zero and covariance matrix as:

$$\left(\begin{array}{c} e_{i}\\ w_{i} \end{array}\right)\sim N_{2}\left[\left(\begin{array}{c} 0\\ 0 \end{array}\right),\left(\begin{array}{cc} \sigma^{2} & \rho \sigma \\ \rho \sigma & 1 \end{array}\right)\right]$$

Under assumptions (3.6) and (3.7), the ATT can be derived following Greene (2012) as:

$$\mathrm{ATT}=E\left(Y_{i}|X_{\mathrm{ji}},Z_{\mathrm{ki}},d_{i}=1\right)-E\left(Y_{i}|X_{\mathrm{ji}},Z_{\mathrm{ki}},d_{i}=0\right)$$

(8) $$=\;\delta +\rho \sigma \;\left[\emptyset \left(\alpha Z_{k}\right)/\Phi \left(\alpha Z_{k}\right)\;\left[1-\Phi \left(\alpha Z_{k}\right)\right]\right]$$

where σ is the standard deviation of the error term wi; $\emptyset \left(.\right)$ denotes the standard normal density function; and $\Phi \left(.\right)$ denotes the standard normal cumulative distribution function assumed for the probit model. The treatment effect can be obtained using maximum likelihood estimation (MLE) or in a two‐step consistent estimation (Maddala 1983). The ATT in Equation (8) reduces to δ whenever ρ equals zero. This implies that the two error terms ei and wi are independent and there is no endogeneity problem (Cameron and Trivedi 2010) that undermines PSM estimates.

#### 2.3.2 The Unrestricted Model Specification

The unrestricted forms of the endogenous treatment effects models such as the endogenous switching regression (ESR) are the generalized forms of the restricted model to the potential outcome framework attributed to Neyman (1923) by Frangakis and Rubin (2002). Following Heckman et al. (2003), the model with separate variance and correlation coefficients for the adopters and non‐adopters can be expressed as:

(9) $$Y_{1i}=\beta_{1j}X_{\mathrm{ji}}+e_{1i}$$

(10) $$Y_{0i}=\beta_{0j}X_{\mathrm{ji}}+e_{0i}$$

(11) $$d_{i}=\left\{\begin{array}{c} 1,\;\text{if}\;\alpha_{k}Z_{k}+w_{i}>0\\ 0,\;\text{otherwise} \end{array}\right.$$

with the assumption that:

(12) $$\mathrm{cov}\left(x_{j},e_{1i}\right)=\mathrm{cov}\left(x_{j},e_{0i}\right)=\mathrm{ov}\left(e_{1i},e_{0i}\right)=0$$

(13) $$\mathrm{cov}\left(w_{i},e_{1i}\right)=\rho_{1}$$

(14) $$\mathrm{cov}\left(w_{i},e_{0i}\right)=\rho_{0}$$

The error terms $e_{1i}$, $e_{0i}$ and $w_{i}$ are also assumed to follow a trivariate normal distribution with covariance matrix:

$$\left(\begin{array}{c} e_{1i}\\ e_{0i}\\ w_{i} \end{array}\right)\sim N_{3}\left[\left(\begin{array}{c} 0\\ 0\\ 0 \end{array}\right),\left(\begin{array}{ccc} \sigma_{1}^{2} & 0 & \rho_{1}\sigma_{1}\\ 0 & \sigma_{0}^{2} & \rho_{0}\sigma_{0}\\ \rho_{1}\sigma_{1} & \rho_{0}\sigma_{0} & 1 \end{array}\right)\right]$$

Under assumptions (3.12), (3.13), and (3.14), the ATT can be expressed as:

$$\mathrm{ATT}=E\left(Y_{1i}|X_{\mathrm{ji}},Z_{\mathrm{ki}},d_{i}=1\right)-E\left(Y_{0i}|X_{\mathrm{ji}},Z_{\mathrm{ki}},d_{i}=1\right)$$

(15) $$={\left(\beta_{1}-\beta_{0}\right)}^{\prime }X+\left(\rho_{1}\sigma_{1}-\rho_{0}\sigma_{0}\right)\;\left[\emptyset \left(\alpha^{\prime }Z_{k}\right)/\Phi \left(\alpha^{\prime }Z_{k}\right)\right]$$

One major concern with the latter IV estimator is that adopters might anticipate gains from the ICVs. This hidden agenda leads to an unobserved selection into adoption (Shahidur et al. 2016), because farmers who end up benefiting more from the ICVs, given their characteristics *X*, may also be more likely to adopt the ICVs. Shahidur et al. (2016) highlighted that, since the instrument *Z* affects adoption, unobserved heterogeneities driving adoption may also affect *Z*, leading to a biased ATT estimate. This limitation was addressed by Imbens and Angrist (1994) with the introduction of the LATE.

#### 2.3.3 Local Average Treatment Effect

The LATE is the expected gain for those induced to receive the treatment through a change in the instruments from *Z*_*k*_ to *Z*_*k*_^+^. The monotonicity assumption identifying the LATE states that the probability of ICV adoption increases as the instrument changes from *Z*_*k*_ to *Z*_*k*_^+^ (i.e., $P\left(d=1|X,Z^{+}\right)>P\left(d=1|X,Z\right)$). Under this assumption and following Shahidur et al. (2016), the LATE can be expressed as:

(16) $$\text{LATE}=\frac{E\left[Y_{i}|P\left(d_{i}=1|X_{j},Z_{k}^{+}\right)\right]-E\left[Y_{i}|P\left(d_{i}=1|X_{j},Z_{k}\right)\right]}{P\left(d_{i}=1|X_{j},Z_{k}^{+}\right)-P\left(d_{i}=1|X_{j},Z_{k}\right)}$$

(17) $$Y_{i}=\beta_{j}X_{j}+\text{LATE}*{\hat{P}}_{i}+e_{\text{LATE}i}$$

where ${\hat{P}}_{i}$ are the predicted probabilities of ICVs adoption conditional on farmers' characteristics *X*_j_ and the instruments *Z*_*k*_. Equation (12) is estimated using a two‐stage approach where a binary choice probit or logit model is used in the first stage to predict the propensity scores (${\hat{P}}_{i}$) which are then used as a proxy instrument (17) to estimate the LATE (Wiredu et al. 2014; Awotide et al. 2013).

## 3 Materials and Methods

### 3.1 Data

We used data collected from cassava farm households (Cassava farm households refer to families or individuals who cultivate cassava as a primary crop on their farms) in the Ashanti and Brong‐Ahafo regions of Ghana. Data was gathered through a structured questionnaire administered between July and August 2014. These two regions are deciduous forest and transitional agro‐ecological zones with a bimodal rainfall pattern. These regions are the primary contributors to the country's cassava production (MoFA 2024; Acheampong et al. 2022). The regions benefit from fertile soils and favorable rainfall patterns, creating optimal conditions for cassava cultivation. We adopted a multi‐stage cluster sampling technique with optimal allocation to determine the number of communities and cassava‐producing households in the regions. We obtained the sampling frame from MoFA, which included a list of all treated and control districts. Three treated districts (the districts were selected because of their favorable conditions for cassava cultivation and known for massive production and receive technologies aimed at improving cassava production in Ghana) per region were then selected for the study, followed by the establishment of a list of all communities in the six districts. The district directors of the Directorate of Agricultural Extension Services of the Ministry of Food and Agriculture (DAES‐MoFA) assisted in identifying the communities that had ICVs demonstration plots and field days. In the six treated districts, the final sample frame consisted of 15 treated and 217 control communities.

To determine the number of survey communities and the minimum sample size per community, we used power analysis following Spybrook et al. (2011). A random selection of 14 communities (eight treated and six controls) and 41 households per community resulted in a total projected sample size of 574 farm households. If constructed ex‐ante, this sample would help identify at 80% power, a minimum detectable 57% increase in cassava productivity, with a 5% margin of error given the expenditures in previous studies and an intra‐district correlation coefficient of 0.01. We surveyed 608 households, 38% of which were female‐headed, and encouraged enumerators to interview as many households as they could, with a minimum of 41 per community. Appendix A. presents the survey locations per region and district, along with the number of households surveyed per type of community.

We commissioned face‐to‐face interviews of farm households, and respondents were randomly selected using a random number table after establishing the list of all households in the community. Eight enumerators, two supervisors, and supra‐supervisors were hired, trained, and involved in the refinement of the sampling and data collection processes to ensure quality assessment. Survey instruments (household listing form and questionnaire) were pretested and improved prior to data collection. The questionnaire comprised modules on households' socioeconomic, institutional characteristics, farming systems information, outputs obtained per crop, and the quantities sold and remaining on the farm. Other modules comprised the knowledge and adoption of local cassava varieties and ICVs, sources of information and reasons for adopting or abandoning the varieties, the cost of inputs and labor, and the main buyers of the output. The headcount and income from livestock owned and farm implements used were also collected with off‐farm incomes and expenditures.

### 3.2 Estimation Variables

Variable definitions and summary descriptives used in the models are presented in Table 1. Following the previous studies on ICVs adoption (e.g., Mwebaze et al. 2024; Awotide et al. 2015; Feleke et al. 2016; Mdoda et al. 2025; Khonje et al. 2015; Tufa et al. 2019; Wossen et al. 2019), we include a set of covariates (i.e., gender, age, education, household size, farm size, land ownership, farm plots, herd ownership, FBO membership, credit access, distance to the nearest tarred road, distance to the local market, extension access, and innovative platform). Location dummies were included to address the unobserved heterogeneities, such as socioeconomic and region‐specific institutional conditions, that may also influence farm households' decisions to adopt ICVs. We followed previous literature (Awotide et al. 2015; González‐Flores et al. 2014; Villano et al. 2015) to identify the pre‐adoption covariates (*X*_j_) used in matching estimators. The instruments (*Z*_*k*_) are based on ICVs dissemination strategies adopted by WAAPP‐Ghana implementing partners.

**TABLE 1:** Definition of estimation variables and summary statistics.

Variables	Notation	Definition	Adopters	Non‐adopters	Mean‐difference	All respondents			
			Mean	Mean		Mean	SD	Min	Max
Outcome variables									
*Crevha*	Y1	Cassava land productivity (GH₵/ha)	2270	1746	524***	1877	1928	37	31,135
*Trevha*	Y2	Whole‐farm land productivity (GH₵/ha)	2012	1586	426***	1692	1465	37	23,957
*Allincp*	Y3	Per capita annual total income (GH₵)	570	494	76	513	908	−2032	11,509
*Agincp*	Y4	Per capita agricultural income (GH₵)	357	324	33	332	678	−2032	9416
*Fincp*	Y5	Per capita annual crop income (GH₵)	179	201	−22	195	552	−2032	9416
*aninc2p*	Y6	Per capita annual income from livestock (GH₵)	178	123	54	137	384	−6	4906
*Offincp*	Y7	Per capita annual off‐farm income (GH₵)	213	169	43	180	557	0	10,225
*Hhexpp*	Y8	Per capita total annual expenditures (GH₵)	843	949	−106	922	728	44	8300
*Foodp*	Y9	Per capita annual food expenditure (GH₵)	429	495	−66*	479	387	5	5210
*Kideducp*	Y10	Annual education expenditures (GH₵/school child)	388	456	−67	437	592	3	6400
Variables in the probit models									
PSM and IV									
*Impvar*	d	1 if household planted one ICV, 0 otherwise (%)				0.25	0.43	0	1
*Female*	X1	1 if household head is female (%)	0.34	0.39	−0.05	0.38	0.49	0	1
*Age*	X2	Age of the household head (years)	47.86	48.29	−0.42	48.18	11.88	20	96
*Educ*	X3	Number of years of formal education by hh head	9.86	8.66	1.19**	8.96	5.76	0	24
*Hhsize*	X4	Household size (#)	6.27	5.87	0.40	5.97	2.63	1	18
*Ashanti*	X5	1 if hh in the Ashanti region, 0 if in Brong‐Ahafo	0.42	0.54	−0.12***	0.51	0.50	0	1
*Areasize*	X6	Total area owned (ha)	2.58	2.42	0.46*	2.51	2.18	0.20	32.37
*Landown*	X7	Land ownership: 1 if owned, 0 if other (%)	0.85	0.88	−0.03	0.88	0.33	0	1
*Plot*	X8	Number of plots farmed (#)	1.55	1.40	0.15**	1.44	0.64	1	6
*plot2*	X9	Square of the Number of plots farmed				2.48	2.65	1	36
*Herd*	X10	Number of sheep equivalent owned (#)	6.57	4.22	2.35	4.80	15.57	0	246
*Fbo*	X11	1 if hh is member of a group, 0 otherwise (%)	0.53	0.22	0.31***	0.30	0.46	0	1
*Credit*	X12	1 if hh has access to credit, 0 otherwise (%)	0.17	0.06	0.11***	0.09	0.28	0	1
troad*distlm	X13	Interaction between troad and distlm				9.57	25.11	0	360
*troad*		Distance to the nearest tarred road (miles)	1.84	2.44	−0.60***	2.29	2.43	0	23.50
*Distlm*		Distance to the local market (miles)	2.09	3.22	−1.13***	2.94	3.87	0.1	25
IV only									
*Ext*	Z1	1 if ICV known through the extension agent (%)	0.89	0.16	0.73***	0.35	0.48	0	1
*Ip*	Z2	1 if ICV known through innovation platforms (%)	0.013	0.002	0.011*	0.01	0.07	0	1

Abbreviation: SD, standard deviation.

* *p* < 0.10.

** *p* < 0.05.

*** *p* < 0.01.

Table 1 presents summary statistics for the sample, disaggregated by adoption status. Adopters and non‐adopters differ significantly. Adopters are younger on average, which may reflect greater openness to new technologies or longer remaining planning horizons over which to recoup adoption costs. This is consistent with the technology adoption literature's finding that age is often negatively associated with adoption (Mwebaze et al. 2024; Asante et al. 2023; Kamara et al. 2025). Adopters also have significantly more years of education than non‐adopters, supporting the common finding that education improves farmers' ability to access, process, and act on information about new varieties (Bosompem et al. 2024; Mwebaze et al. 2024; Asante et al. 2023). Most households own their land, over a third are female‐headed, and average landholding is just over two hectares spread across multiple plots. Average household size (6) exceeds the national average (4) (GSS 2021), which may reflect the labor‐intensive nature of cassava cultivation. Adopters operate significantly larger landholdings than non‐adopters, consistent with land area being both a resource for and an outcome of adoption decisions.

**FIGURE 1:**
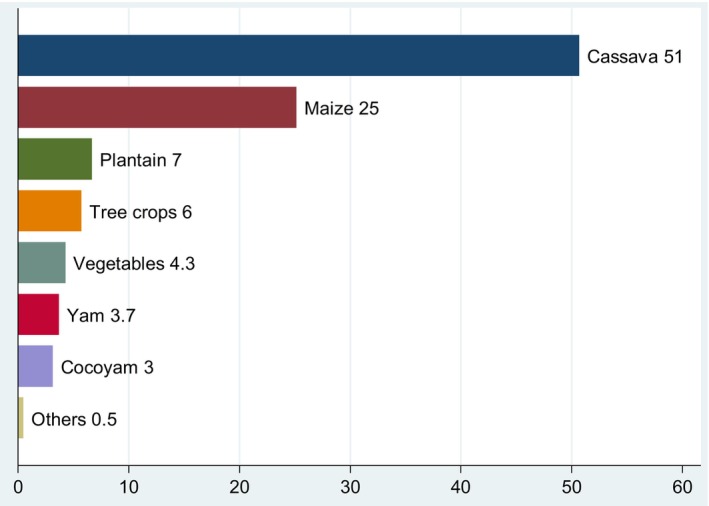
Share of total crop revenue in percentage.

The results show that if farm households were to trade all their produce, they would have obtained, on average and per hectare of land cultivated, an estimate of GH₵ 1877 for cassava and GH₵ 1692 for all crops produced. The first (*Y*_1_) is measured as the value of cassava output (revenue) divided by the area planted to cassava, while the latter (*Y*_2_) is the value of all crops produced divided by the total area cultivated. The results also show that revenue from cassava makes up more than half of the total revenue derived from all crops produced, while the share of maize is 25% (Figure 1). Revenues from plantains represent 7% of the total crop revenue. The tree crops (cocoa, coffee, rubber, and others) account for 6% of the whole‐farm revenue, and the mean per capita annual expenditures amount to GH₵ 922. Per capita total annual income (*Y*_3_) is the aggregated income from agricultural and off‐farm activities divided by household size. The per capita annual agricultural income (*Y*_4_) is the sum of the per capita income from all crops planted (*Y*_5_) and the per capita income from livestock farming (*Y*_6_). We measured the income from livestock (Y_6_) as the value of all owned livestock (ruminants, pigs, and poultry) minus the production costs. Per capita annual off‐farm income (*Y*_7_) is the aggregate incomes from salaries, property rental, commerce, artisanal activities, pension, insurance, remittances, and other incomes earned by the household divided by the household size.

**FIGURE 2:**
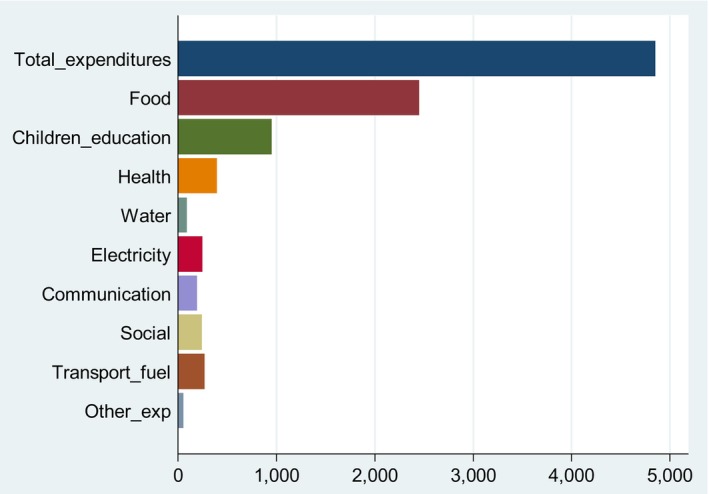
Household mean expenditures in GHC in 2013.

Total annual expenditure per capita (*Y*_8_) is the sum of annual expenditures on food, children's education, health, water, electricity, communication, social activities, fuel, and other expenses divided by the household size. We further disaggregated the total annual expenditures per capita by the per capita expenditures on food (*Y*_9_) and expenditures by children in school (*Y*_10_) because expenses on food and children's education are the largest proportions of total expenditures, as shown in Figure 2.

### 3.3 Estimation Techniques

This study aims to estimate the impact of ICVs on the productivity and welfare of farm households in Ghana. This section focuses on the empirical procedures used and the results of the tests conducted to select the most suitable impact estimators.

#### 3.3.1 Choice of the Appropriate Matching Technique

**FIGURE 3:**
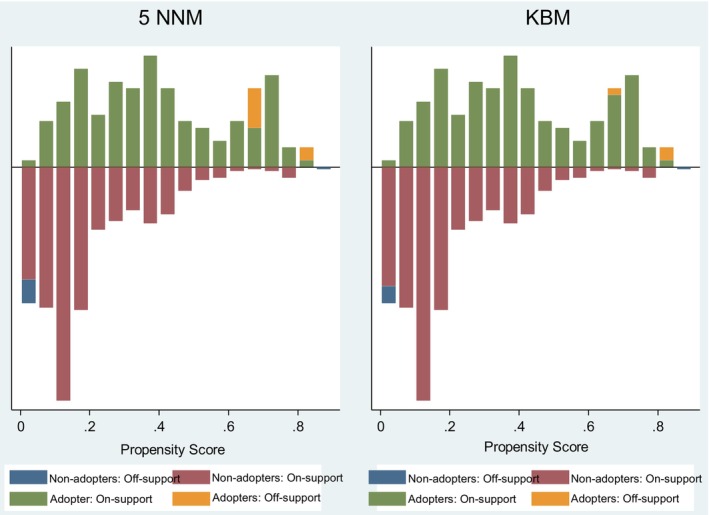
Common support regions with the 5‐nearest neighbor and kernel based matchings.

Following Leuven and Sianesi (2003), we estimated the ATT for the six PSM techniques and proceeded with the series of balancing tests discussed in Section 2.2. To ensure more rigorous matching, we imposed a caliper width (maximum distance of non‐adopters) of 0.025 on the nearest neighbor (Guo and Fraser 2014). We also utilized the RM for a more rigorous tolerance distance of 0.0011. We adopted the default Epanechnikov kernel type for KBM and the LLRM. The results presented in Appendix B favor the 5‐NNM, which displays the smallest pseudo‐*R* ^2^, *χ* ^2^, and remaining mean and median bias as well as Rubin's *B* and *R* compared with the KBM. The test statistics of the mean difference in the covariates for adopters and non‐adopters after matching (t‐af) were still significant for the variable “fbo” (*p* < 0.05) under the 1‐to‐1 NNM without replacement. The test statistic was also significant for three covariates after the 1‐to‐1 NNM with replacement and for four covariates after the LLRM, for which the *p* > *χ* ^2^ was significant (*p* < 0.10) after matching, implying failure of the balancing proper. The 5‐NNM and the KBM, which best satisfy the balancing properties, resulted respectively in 20 and 12 to be off‐support. This resonates with the findings of González‐Flores et al. (2014) and Villano et al. (2015). The density estimates of the distribution of the propensity scores for each of the adopters and non‐adopters, as well as the regions with and without support, are shown for the 5‐NNM and the KBM in Figure 3.

We conducted the default Mahalanobis NNM on observables with bias correction for matching on multiple continuous variables (Abadie and Imbens 2011) to test the robustness of the PSM estimator. For this purpose, we estimated the ATT using the *effects nnmatch* command with bias adjustment option built in *Stata* (StataCorp 2015b) and imposed a number of five matches per observation.

#### 3.3.2 Choice of the Appropriate Specification of the Endogenous Treatment‐Effect Regression

To assess the impact of ICV adoption on various outcome indicators, including cassava land productivity, whole‐farm land productivity, various per capita income and expenditures measures, we estimated a counterfactual scenario. Using endogenous treatment effects to analyze the adoption of ICVs on welfare is essential for several reasons. First, ETR addresses selection bias, ensuring a more accurate estimation of the impact of ICV adoption and further account for unobserved variables that may influence both adoption and outcome indicators. We assumed that no unobserved characteristic influencing these outcomes would also affect adoption decisions. We used the CIA to match adopters with non‐adopters based on propensity scores (Imbens and Wooldridge 2009; Wooldridge 2010). We then fitted the full Maximum Likelihood Estimation (MLE) of both restricted and unrestricted endogenous treatment effects models, as well as the LATE. To distinguish between the restricted and generalized forms, we applied the generalized LR‐test, which tested the null hypothesis that the restricted model nests within the unrestricted one (see Appendix C). After selecting the appropriate specification model based on the LR‐test results, we tested for the adoption endogeneity. This was done using a Wald test to check the significance of the disturbances' correlation coefficient $\mathrm{rho}\;\left(\rho \right)$ (Terza et al. 2008). If ρ is significant, the IV estimator is preferred; otherwise, the PSM estimator is deemed appropriate.

In addition, we applied the Durbin and Wu–Hausman test of endogeneity within a two‐stage least square regression framework for continuous treatment. The result validated the two instruments used. We also conducted Hansen's test of overidentifying restriction, which was not significant [*χ* ^2^(3) = 2.99977 (*p* = 0.3917)]. This validates the instruments' lack of correlation with the error term and their correct exclusion from the outcome equation.

## 4 Empirical Results and Discussion

### 4.1 Impact of the Adoption of ICVs on Land Productivity

**FIGURE 4:**
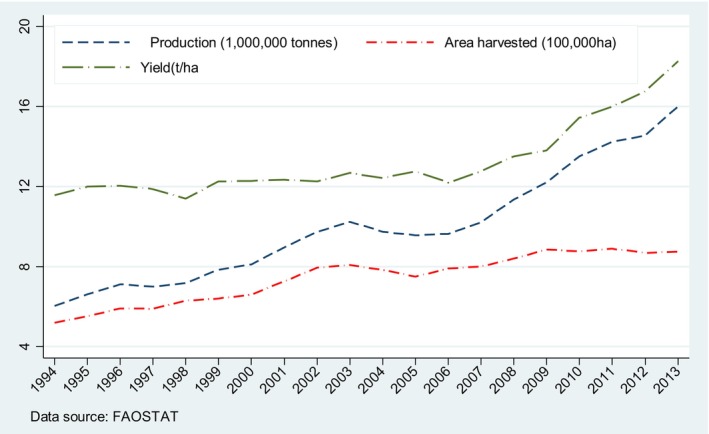
Trend in cassava area harvested, production, and yield in Ghana.

Table 2 summarizes the impact of ICVs adoption as estimated through matching and IV procedures. We also report the standard errors for the PSM estimators, obtained through 50‐repetition bootstrapping. The last column of Table 2 indicates the appropriate estimator, determined by the *p*‐value of the Wald‐test. This test checks for no correlation between the error terms in the adoption and outcome models, as reported by the full MLE. We derive the ATTs of the IV estimator from the suitable specification of the endogenous treatment effects regressions (Appendix C). This suggests that the unconstrained potential outcome specification aptly represents the data for the outcome variables where the IV was most suitable. The findings highlight a positive impact of ICVs adoption on land productivity, with slight variations depending on the matching estimator used. Holding all other factors constant, planting an ICVs significantly boosts cassava land productivity by between GH₵ 490/ha and GH₵ 512/ha and whole‐farm productivity by GH₵ 371/ha GH₵ 398/ha for the adopters. If we had erroneously considered only the unsuitable IV estimates for the productivity indicators, we would have inaccurately reported that ICVs have no impact on land productivity. These findings align with previous studies on the impacts of agricultural technology adoption on productivity (Kolapo and Kolapo 2021; Acheampong et al. 2022; Tufa et al. 2019; Wossen et al. 2019). Furthermore, as depicted in Figure 4, these findings are consistent with trends in FAOSTAT (2015) historical cassava production and harvested areas.

**TABLE 2:** Impact of ICV adoption on land productivity and household income and expenditures.

Welfare indicator	5NN‐PSM	KB—PSM	Matching on observables	IV Estimator	LATE	Endogeneity test (H_0_: $\rho =0\;\text{or}\;\rho_{1}=\rho_{0}=0$)^a^	
	ATT	ATT	ATT	ATT		*p*	Better estimator
Land productivity (GH₵/ha)							
Cassava productivity	490*** (210)	512*** (135)	505*** (142)	372 (232)	240 (306)	0.440	Matching
Whole‐farm productivity	371*** (157)	381*** (148)	398*** (133)	281 (184)	248 (233)	0.651	Matching
Per capita annual incomes (GH₵/capita)							
Per capita total annual income	104 (106)	115 (118)	55 (67)	16 (142)	28 (126)	0.805	Matching
Per capita agricultural income	42 (119)	50 (91)	−9 (50)	92 (118)	60 (90)	0.402	Matching
Per capita crop income	41 (72)	44 (77)	27 (41)	148* (80)	41 (79)	0.000	IV
Per capita livestock income	1 (104)	6 (70)	−37 (32)	43 (76)	18 (45)	0.498	Matching
Per capita off‐farm income	62 (56)	65 (54)	64 (45)	−15 (87)	−32 (87)	0.569	Matching
Annual per capita expenditures							
Total expenditures (GH₵/capita)	−117* (72)	−90 (71)	−94 (69)	−126 (91)	−55 (106)	0.804	Matching
Food (GH₵/capita)	−66 ** (36)	−52* (34)	−28 (32)	−145*** (47)	−126** (55)	0.038	IV
Children's education (GH₵/child in school)	−72 (83)	−64 (56)	−108 (70)	172* (100)	242** (96)	0.001	IV

*Note:* Standard errors in parenthesis.

^a^ IV is preferred when H_0_ is rejected.

* *p* < 0.10.

** *p* < 0.05.

*** *p* < 0.01.

Contrasting with the findings of Garrity et al. (2012), trends in Figure 4 show a steeper increase in production and productivity per hectare from 2006 and a slow growth in the areas harvested. This could imply that the increases in production from 2006 stemmed more from gains in productivity than from the expansion of areas planted with cassava.

### 4.2 Impact of ICV Adoption on Household Welfare Indicators

The results presented in Table 2 show that ICVs adoption has no significant impact on the overall per capita annual income. A closer look at the disaggregated income sources, however, reveals that the unrestricted IV estimator provides a positive and significant impact of ICV adoption on annual crop income per person. On average, adopting ICVs results in an increase of GH₵148 per capita in crop incomes. This finding is in line with Acheampong et al. (2022). As seen in earlier research (Ahimbisibwe et al. 2023; Acheampong et al. 2022; Khonje et al. 2015; Wossen et al. 2019), there is a positive relationship between productivity, incomes, food security, and poverty reduction. These results suggest that using ICVs could improve food security and reduce poverty. This is largely due to cassava being a primary source of calorie intake for households and a significant contributor to crop incomes and household expenditures, with about 50% being spent on food and 20% on children's education. Interestingly, we found no significant impact on incomes derived from livestock and off‐farm sources. This suggests that ICVs adopters may not redirect their incomes from these sources into cassava production. Additionally, the results show that ICVs adopters spend, on average, GH₵117 less per capita per year than non‐adopters. This negative impact is consistent across all estimators, though it is only significant for the most efficient 5‐NNM.

To enrich the understanding of ICV impacts, the study disaggregated the analysis by expenditure on food and children's education. The findings reveal that adopters significantly spend less on food and more on children's education than non‐adopters. The reduction in annual food expenditures is GH₵ 145/capita for the adopters and GH₵ 126/capita for those induced to adopt the ICVs through varietal promotion activities. For the latter group, adoption of ICV results in significant increases in children's education expenses per child in school and per year. Possible explanations of these findings are that adopters enjoying higher cassava productivity might use part of their harvest to offset household food requirements and could invest the expenses saved on food in children's education. These findings correspond with previous findings (Feleke et al. 2016; Wossen et al. 2019), who found positive effects of the adoption of improved cassava varieties on incomes and poverty in Africa.

Moreover, the interaction between the adoption and gender indicator variables in the potential outcome specification of the IV model is presented in Table 3. Looking at results for important outcome variables, adopting an ICV does not change the per capita crop income or food spending of female‐headed households that adopt it (Table 3). Female‐headed households growing local varieties do, however, experience GH₵ 81 per capita reductions in food expenses compared to their male counterparts. These findings imply that female household heads use more of their local varieties grown for household consumption than male‐headed households. In addition, the adoption has no impact on expenditure on children's education for the female‐headed adopters. However, those who grow the local varieties contract more debt (approximately GH₵ 154 per year for a child sent to school) than their counterpart male non‐adopters. Cumulatively, the results imply that promotion of ICV adoption and other income generation activities will not only increase productivity and crop income among adopters but may financially empower female‐headed households for increased investment in household consumption and children's education.

**TABLE 3:** Dissagregated analysis of female‐headed households' income.

Indicator	Population	Adoption status of female headed household	
		Local	Improved
	ATT	ATT	ATT
Per capita crop income	148 (119)	−28 (36)	−32 (57)
Food (GH₵/capita)	−143*** (50)	−81** (33)	−41 (42)
Children's education (GH₵/child in school)	170 (143)	−154*** (57)	29 (65)

*Note:* Standard errors in parenthesis.

** *p* < 0.05.

*** *p* < 0.01.

## 5 Conclusion and Policy Recommendations

This study examined the impact of Improved Cassava Varieties (ICV) adoption on specific farm performance and livelihood indicators. It utilized observational data obtained from a random sample of 608 cassava‐producing households in six districts located in two regions of Ghana. The study applied Propensity Score Matching (PSM), Local Average Treatment Effect (LATE), and endogenous treatment effects regression methods to estimate the effects of ICV adoption. These methods helped us account for biases caused by both observable and unobservable characteristics.

The summary statistics indicate a 25% ICV adoption rate. The data suggest that ICV adoption positively impacts cassava yield, overall land productivity, and per capita annual crop income. Interestingly, the findings show that adopters tend to have lower total annual per capita expenditures and food expenditures compared to non‐adopters. The plausible explanation is that lower food expenditure may reflect improved food self‐sufficiency, where increased cassava productivity enables households to rely more on own‐produced food and reduces the need for market purchases. Given that ICV adoption is also associated with productivity and income outcomes, the expenditure results likely reflect changes in household food acquisition patterns. However, adopters invest more in children's education. Increased educational investment suggests that adoption may allow households to reallocate resources toward longer‐term human capital development. Notably, female‐headed non‐adopting households incur higher debts from educating their children compared to their male counterparts. Female‐headed households may face different financial constraints and expenditure priorities, including greater reliance on household resources for food and education‐related expenses. The finding that female‐headed non‐adopters experience higher education‐related debt suggests that limited access to productivity‐enhancing technologies may increase financial pressure among vulnerable households.

Building on the study's findings, the following actionable policy recommendations are proposed. Given that female‐headed non‐adopting households carry significantly higher education‐related debt, programs should prioritize female‐headed households for ICV access through targeted planting material distribution, women‐focused farmer groups and innovation platforms, and female extension agents or community based advisors where cultural norms limit women's interaction with male agents. Bundling ICV access with small‐scale credit or input subsidies could help offset the upfront costs that may otherwise deter adoption among credit‐constrained female‐headed households. With only few farmers having access to credit and EAs serving as the dominant information channel, mobile‐based extension platforms such as SMS or app‐based agronomic advice, weather alerts, and market price information could extend EA reach at lower marginal cost, particularly in regions where in‐person EA visits are infrequent. Low credit access and FBO membership suggest that adoption gains could be amplified by linking ICV dissemination to credit products designed for input purchases, and by strengthening FBOs as channels for both information dissemination and group‐based lending.

While the study offers valuable insights into the relationship between ICV adoption, productivity, and welfare, several limitations warrant discussion. First, the cross‐sectional nature of the data limits causal interpretation and precludes analysis of the temporal dynamics of ICV adoption. Adoption is a process that unfolds over time. Early adopters may accumulate experience, refine management practices, and reinvest productivity gains in ways that amplify welfare impacts, while late adopters may not yet have realized the full benefits at the time of survey. A single cross‐section cannot capture these trajectories, meaning the estimates reflect average effects at a particular point in adoption history rather than long‐run impacts. Panel data would allow identification of adoption timing effects and dynamic complementarities between ICV use and other inputs, and future research should prioritize this approach. Second, income and expenditure data collected through recall surveys are subject to measurement error. Respondents may underreport irregular income sources, misremember seasonal expenditures, or anchor responses to salient but unrepresentative periods. In smallholder agricultural settings, where income is lumpy and seasonal, such recall bias can introduce attenuation bias in outcome measures, potentially understating the true welfare effects of ICV adoption. The study mitigates this concern partly through the use of multiple welfare indicators such as productivity, income, and expenditure, which are unlikely to be biased in the same direction simultaneously, lending some robustness to the overall pattern of findings. Third, the dataset does not include distance to demonstration plots, as farmers faced difficulty estimating distances accurately. Future studies should consider objective distance measurements as an additional instrument, which may strengthen identification and allow sensitivity analysis of the exclusion restriction.

## Funding

The study was conducted with funding support from the West and Central African Council for Agricultural Research Development (CORAF) and the Australian Department of Foreign Affairs and Trade (DFAT).

## Disclosure

AI contribution: The authors declare that artificial intelligence (AI) was not utilized in this study.

## Ethics Statement

This study received ethical approval from the Research Committee of the Business School at the University of New England, Australia. Prior to data collection, informed consent was obtained from all participating farmers, who were informed of the study's purpose, the voluntary nature of their participation, and their right to withdraw at any time without penalty. Confidentiality of respondent information was maintained throughout the data collection and analysis process.

## Conflicts of Interest

The authors declare no conflicts of interest.

## Supporting information


**Data S1:** pei370186‐sup‐0001‐Supinfo.dta.

## Data Availability

The data supporting the findings of this study are available in the Supporting Information file named cassava_dta_050815.dta.

## Appendix A Survey Locations and Sample Size

**Table:**

Region	District	Community	Farm households surveyed	
			Expected	Achieved
Ashanti	Afigya Krabe	Abedease‐Kyekyewere^a^	41	43
		Amoako	41	53
	Atwima Kwanwoma	Afrancho^a^	41	43
		Foase^a^	41	46
		Nweneso	41	47
	Sekyere Central	Bonkrong^a^	41	36
		Gyansa	41	41
Brong Ahafo	Asutifi	Mehame^a^	41	41
		Wuramumuso	41	41
	Sunyani West	Kwatire	41	45
		Nsoatre^a^	41	43
	Wenchi	Awisa	41	43
		Subinso^a^	41	43
		Nkonsia^a^	41	43
Total	6	14	574	608 (106%)

^a^ Treated communities.

## Appendix B Balancing Property Diagnosis and Selection of the Appropriate Matching Technique

**Table:**

	Unmatched sample		1‐to‐1without replacement		1‐to‐1 with replacement		5‐NN matching		Radius matching		Kernel matching		LLR matching	
	t_bf^a^	Vt/Vc^b^	t‐af^c^	Vt/Vc	t_af	Vt/Vc	t‐af	Vt/Vc	t‐af	Vt/Vc	t‐af	Vt/Vc	t‐af	Vt/Vc
Covariates														
*female*	−1.09	0.94	0.91	1.03	−0.37	0.97	0.08	1.01	0.63	1.05	−0.30	0.95	−0.61	0.94
*age*	−0.38	0.94	0.98	0.85	1.87c	1.01	0.6	0.94	0.43	0.79*	0.63	1.02	2.12b	1.03
*educ*	2.22b	1.05	0.11	1.30*	0.01	1.21	−0.35	1.29*	−1.66	1.23	0.06	1.14	0.31	1.18
*hhsize*	1.61	0.81	0.19	0.91	0.59	0.80	−0.27	0.8	0.20	0.62*	−0.16	0.83	0.58	0.78*
*ashanti*	−2.59a	1.00	−0.30	1.03	−1.78c	0.89	−0.95	0.77*	−0.89	1.25	−1.25	0.77*	−1.87c	0.89
*areasize*	1.89c	0.48**	0.10	0.65*	2.07b	1.16	−0.07	0.60*	1.29	0.92	0.28	0.62*	2.09b	1.07
*landown*	−0.96	1.22	0.95	0.80	1.53	0.77*	0.54	0.97	−0.19	1.05	0.92	0.91	1.81c	0.72*
*plot*	2.43b	0.61*	−1.06	0.95	−0.40	0.86	0.16	0.89	−1.43	0.86	0.56	0.81	−0.20	0.85
*plot*plot*	1.26	0.41**	−0.92	1.01	−0.61	0.72*	0.07	0.86	−1.52	0.69*	0.51	0.83	−0.44	0.71*
*herd*	1.61	1.38*	1.61	1.38*	−0.24	0.50**	−0.41	0.52*	0.48	1.58*	−0.36	0.56*	−0.24	0.50**
*fbo*	7.44a	0.86	−0.85b	0.38**	0.24	0.97	0.02	0.99	0.04	0.98	−0.26	1.10	0.12	0.98
*credit*	4.33a	2.64**	−2.15	0.93	−0.92	0.81	−0.79	0.71*	1.38	1.55*	−0.78	0.78*	−1.03	0.80
*troad*distlm*	−3.38a	0.13**	−0.86	0.75*	0.19	1.68*	−0.11	0.8	0.20	1.23	−0.45	0.71*	0.21	1.68*
Indicators														
Pseudo *R* ^2^	0.192		0.071		0.046		0.006		0.048		0.009		0.051	
LR *χ* ^2^	130.74		17.38		18.23		2.44		10.34		3.76		20.86	
*P* > *χ* ^2^	0.000		0.182		0.149		0.999		0.666		0.993		0.076	
Mean bias	22.6		11.9		9.7		4.3		12.1		5.8		10.3	
Median bias	19.0		13.5		6.4		3.4		9.9		4.6		6.8	
Rubin's *B* ^d^	96.1*		65.7*		51.3*		18.4		52.1*		22.6		54.0*	
Rubin's *R*	0.35*		1.37		1.12		1.09		0.55		1.34		1.31	
% concern	11		22		17		28		28		28		22	
% bad	22		6		6		0		0		0		6	
*N*‐control	457		121		445		445		149		448		448	
*N*‐treated	151		119		143		143		77		148		148	
*N*‐total	608		240		588		588		226		596		596	

^a^ t_bf is the test statistic of the *t*‐test of mean difference in the covariates between treated and control observations before matching. Significance levels a 1%; b 5%; c 10%.

^b^ Vt/Vc for the matched sample: * if “of concern”, that is, variance ratio in [0.5, 0.8) or (1.25, 2]; ** if “bad”, that is, variance ratio < 0.5 or > 2.

^c^ t_af is the test statistic of the *t*‐test of mean difference in the covariates between treated (adopters) and control (non‐adopters) observations after matching. Significance levels a 1%; b 5%; c 10%.

^d^ Rubin's (2001) *B* and *R*: * if *B* > 25%, *R* outside [0.5; 2].

## Appendix C Selection of the Appropriate Endogenous Treatment‐Effects Regression

**Table:**

Indicator	Restricted IV		Unrestricted IV		Likelihood ratio test (H_0_: Restricted nested in unrestricted)		
	ATT	Log likelihood	ATT	Log likelihood	LR‐stat^a^	Decision	Appropriate specification
Land productivity (GH₵/ha)							
Cassava productivity	307.65	−5620.00	372.47	−5601.00	38.01	Reject H_0_	Unrestricted
Whole‐farm productivity	295.88	−5454.40	281.02	−5435.79	37.21	Reject H_0_	Unrestricted
Per capita annual incomes (GH₵/capita)							
Per capita total annual income	16.06	−5080.99	18.88	−5077.02	7.95	Do not reject H_0_	Restricted
Per capita agricultural income	92.11	−4878.39	206.86**	−4875.12	6.53	Do not reject H_0_	Restricted
Per capita crop income	56.56	−4802.68	147.70*	−4793.54	18.27	Reject H_0_	Unrestricted
Per capita livestock income	43.39	−4454.07	46.31	−4451.05	6.05	Do not reject H_0_	Restricted
Per capita off‐farm income	−12.16	−4855.32	−15.33	−4850.11	10.42	Reject H_0_	Unrestricted
Annual per capita expenditures							
Total expenditures (GH₵/capita)	884.04***	−4962.43	−125.50	−4939.43	46.00	Reject H_0_	Unrestricted
Food (GH₵/capita)	−135.00***	−4580.25	−144.82***	−4558.88	42.75	Reject H_0_	Unrestricted
Children's education (GH₵/child in school)	593.46***	−4262.18	172.20*	−4240.59	43.17	Reject H_0_	Unrestricted

*Note:* Critical value obtained from chi‐square distribution table for 5 degrees of freedom 9.24 for *p* < 0.10, 11.07 for *p* < 0.05 and for 15.09 *p* < 0.01.

^a^ LR‐Stat = Likelihood ratio statistics = −2 * [log likelihood restricted – log likelihood unrestricted].

* *p* < 0.10.

** *p* < 0.05.

*** *p* < 0.01.
